# A standardized linear-stapled side-to-side anastomotic technique in robot-assisted Ivor Lewis esophagectomy: a retrospective analysis of 350 consecutive cases from a prospective registry

**DOI:** 10.1007/s11701-025-03132-4

**Published:** 2026-01-24

**Authors:** A. Zeyara, E. A. Kouwenhoven, H. T. J. Mantel, M. J. van Det

**Affiliations:** 1 Department of Surgery, ZGT Hospital, Almelo, The Netherlands; 2 Department of Surgery, Skane University Hospital, Lund, Sweden

**Keywords:** Robotic-assisted minimally invasive esophagectomy, Ivor lewis, Linear stapled side-side anastomosis, Anastomotic complication

## Abstract

Early experiences with robotic-assisted esophagectomy showed disappointing anastomotic outcomes when techniques were directly translated from open or thoracoscopic surgery. Among intrathoracic anastomotic options in robot-assisted Ivor Lewis esophagectomy (RAMIE-IL), emerging data suggest lower complication rates with the linear stapled side-to-side technique. This method was adopted at our institution in 2018. Here, we present our outcomes and describe our standardized linear-stapled side-to-side anastomosis for RAMIE-IL. The primary objective of this study was to detail the surgical technique and evaluate postoperative outcomes of a linear-stapled side-to-side anastomosis in RAMIE-IL. This retrospective cohort study used prospectively collected registry data of all consecutive patients undergoing RAMIE-IL with an intrathoracic linear-stapled side-to-side anastomosis at ZGT Almelo, The Netherlands. All patients from inception (July 1st, 2018) until November 15th, 2025, were included. Data were obtained from the Dutch Upper GI Cancer Audit (DUCA) registry. 350 consecutive patients were included. Mean age was 66 years, 70% were male and 44% had an ASA-class of 3 or more. Length of ICU stay was median 1 days. Length of hospital stay was median 7 days. Overall anastomotic leak rate was 4%. Overall rate of all Clavien-Dindo *≥* 3 complications was 14%. Readmission rate within 30 days was 12.5%. 30-day mortality rate was 1.7%. The presented linear-stapled side-to-side anastomotic technique is feasible and associated with a low rate of anastomotic complications in the studied cohort.

**Supplementary Information:**

The online version contains supplementary material available at 10.1007/s11701-025-03132-4.

## Introduction

Despite increasing centralization and advances in minimally invasive techniques and perioperative care, esophagectomy remains associated with significant postoperative morbidity [1, 2].

Since the implementation of robotic surgery for esophagectomy, with its combined benefits of surgical precision and minimal invasiveness, many anastomotic techniques were directly translated from open or thoracoscopic techniques and initial results for anastomotic outcomes were disappointing [3–7]. Anastomotic complications pose a particular threat to both short- and long-term outcomes, and beyond patient factors and perioperative care, they are closely related to the learning curve of both the center and the individual surgeon, as well as to technical aspects [8]. Anastomotic techniques described for robot-assisted Ivor Lewis esophagectomy (RAMIE-IL) include handsewn (end-to-side), circular stapled (end-to-side) and linear-stapled (side-to-side). Accumulating data is pointing towards lower rates of anastomotic complications with the linear stapled side-to-side technique, and it was therefore implemented in our institution in mid 2018 [3–7]. It was already being used for bariatric surgery and for esophago- and gastrojejunostomies following total and subtotal gastrectomies in our institution.

The aim of this study is to present the outcomes and share our technique of the linear-stapled side-to-side anastomosis in RAMIE-IL with the community.

## Methods

### Manuscript preparation

The manuscript was prepared using the Strengthening the Reporting of Observational Studies in Epidemiology (STROBE) statement [9].

### Study design

Retrospective cohort study from prospectively collected registry data.

### Data collection

This study was based on prospectively collected data from the Dutch Upper GI Cancer Audit (DUCA) registry. All patients aged 18 and above that underwent a RAMIE-IL with an intrathoracic linear-stapled side-to-side anastomosis at the ZGT Hospital in Almelo, the Netherlands, from inception (July 1 st, 2018) until November 15th, 2025, were eligible for inclusion and were assessed for outcomes. All esophagectomies other than robotic Ivor Lewis with an intrathoracic linear-stapled side-side anastomosis were excluded. An absolute majority of all esophagectomies (> 95%) in our institution are performed with this technique.

### Missing data

Missing data were limited (< 5%) and were handled by complete-case analysis; no imputation was performed.

### Study objectives

The primary objective of this study was to detail the surgical technique and evaluate postoperative outcomes of a linear-stapled side-to-side anastomosis in RAMIE-IL. Postoperative outcomes included length of hospital stay (LOS), length of intensive care unit (ICU) stay, 30-day readmission, and 30-day mortality. Procedure-related morbidity was assessed by the incidence of anastomotic complications and by overall Clavien–Dindo grade *≥* III complications. In addition, to ensure comparability with international quality standards, we evaluated achievement of a textbook outcome, defined according to the 2021 international consensus criteria, as meeting all of the following: curative-intent resection; absence of intraoperative complications; microscopic R0 resection with ≥ 15 lymph node yield; absence of serious postoperative morbidity; no reinterventions; no unplanned ICU readmission; no prolonged hospitalization (> 21 days); no 30-day mortality; and no readmission within 30 days of discharge [10].

### Definitions

Postoperative complications were classified according to the Clavien-Dindo classification of surgical complications [10]. 

Anastomotic leaks were defined and graded according to the Esophagectomy Complications Consensus Group (ECCG) definition [11]. 

### Follow-up

All patients (except for a small number included in November 2025) were assessed for 30-day readmission. For patients readmitted within 30 days, granular data were collected to determine whether an anastomotic leak had occurred after discharge. Any anastomotic leak identified during readmission was subsequently recorded in the patient’s anastomotic leak variable.

### Statistics

After assessment of data normality continuous variables were presented as either means (with standard deviations, ±SD) or medians (with interquartile ranges, IQR).

All statistical analyses were performed using the IBM SPSS Statistics 30.0 (Build 172) software for Mac. All graphs were made using Matplotlib for Python.

### Surgical technique

#### General surgical technique for RAMIE-IL in our institution

RAMIE-IL in our institution is performed using the Da Vinci Xi platform (Intuitive Surgical, Inc., Sunnyville, CA, USA) with a four-arm configuration in both the abdominal and thoracic phases. The abdominal phase is performed in French position in a slight anti-Trendelenburg positioning. The lesser curvature is opened, and the hiatus is dissected circumferentially. The greater curvature is opened to the left crus. The pyloric region is dissected with great care to not injure the pedicle of the right gastroepiploic artery, and a semi-Kocher maneuver is performed. Routine lymphadenectomy is performed and include positions 1 and 2 (paracardial), 7 (left gastric), 8 (common hepatic), 9 (celiac trunk), 11p (splenic) and 12a (hepatoduodenal ligament). The right gastric artery is preserved down to the “crow’s foot”. The gastric conduit is constructed with a diameter of about 4.5 cm using the robotic SureForm™ 60 mm stapler. Lastly a pyloromyotomy is performed and a feeding jejunostomy tube is placed. The thoracic phase is performed in semiprone crawl position. After trocar placement, a paravertebral catheter is placed under direct vision. The pleura is incised, and the azygos vein is divided. Then, the esophagus is mobilized in the pericardial and aortic planes, leaving the thoracic duct intact. Routine lymphadenectomy include positions 9 (inferior pulmonary ligament), 8 (paraesophageal) and 7 (subcarinal) whereas positions 2, 4 (paratracheal) and 10 (aortopulmonary window) were only harvested if there were signs of metastases on the preoperative work-up. Lastly, the hiatus is accessed, and the gastric conduit is advanced to the chest.

### Anastomosis

**Fig. 1:**
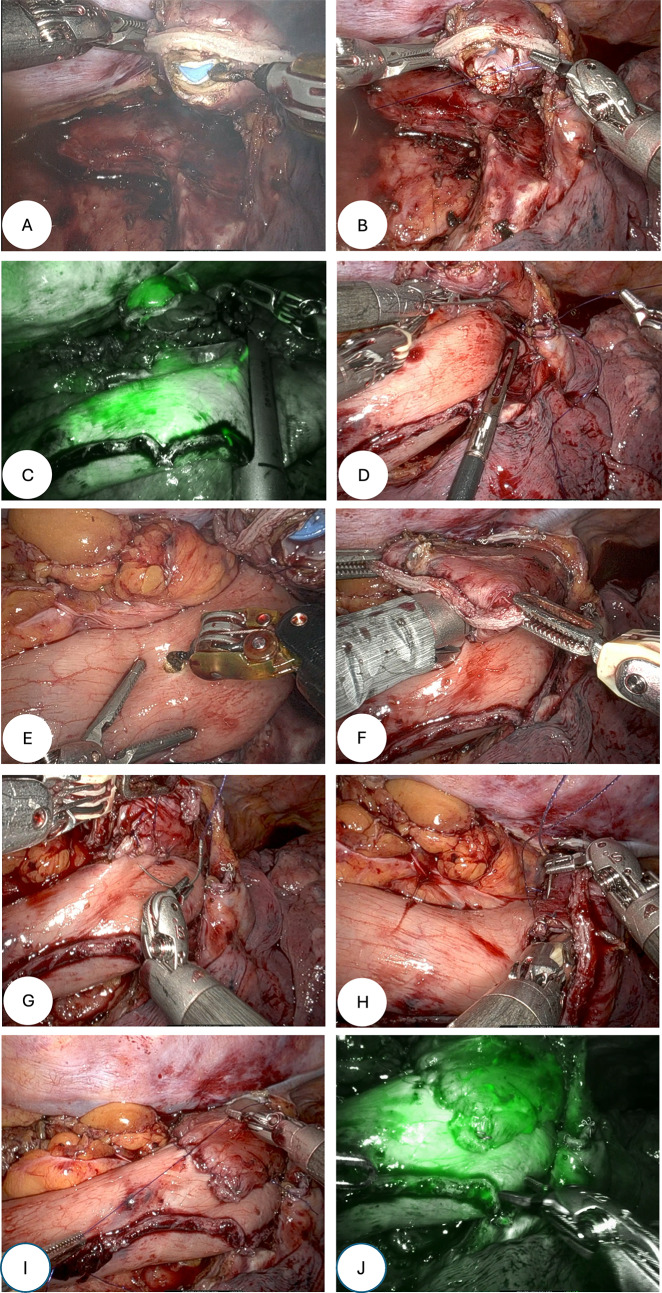
Stepwise construction of the anastomosis. (A) The transected esophagus is elevated using an oral bougie and opened with a cautery hook on the posterior aspect of the staple line. (B) Three 4-0 PDS™ II monofilament sutures a replaced at the 3, 6, and 9 o’clock positions to anchor the mucosa. (C) Indocyanine green (ICG)fluorescence is used to assess conduit perfusion and determine the optimal level of division, ensuring the presence of a feeding artery proximal to the staple line. (D) The gastric conduit is sutured to the pleural edge above the divided azygos vein with 4-0 PDS™ II monofilament sutures. (E) A corresponding gastrotomy is created on the conduit, slightly toward the arcade. (F) A 30-mm robotic stapler with a blue reload cartridge is introduced to fashion the anastomosis. (G, H) The remaining defect is closed with two opposing 4-0 V-Loc™ barbed sutures. (I) The entire suture line is reinforced with two continuous 4-0 PDS™ II monofilament sutures. (J) A final ICG injection confirms adequate perfusion of the completed anastomosis

The transected esophagus is erected by means of an oral Boogie and incised with the cautery hook on the backside of the staple line (Fig. 1A). Three 4 − 0 PDS™ II monofilament sutures (Ethicon, Johnson & Johnson MedTech) sutures are placed to anchor the mucosa, at 3, 6 and 9 o’clock (Fig. 1B). Adequate length of the gastric conduit is measured under mild tension for a straight course and unobstructed drainage towards the duodenum. By means of indocyanine green (ICG; Verdye^®^, Diagnostic Green GmbH, Aschheim-Dornach, Germany) (Fig. 1C) a proper place to divide it is established, as to always have a feeding artery just before the level of the staple line. The gastric conduit is then sutured to the pleural edge (Fig. 1D) above the divided azygos vein with 4 − 0 PDS™ II monofilament sutures (Ethicon, Johnson & Johnson MedTech). A corresponding tomy is made on the gastric conduit (Fig. 1E) slightly more on the side of the arcade and a 30 mm EndoWrist^®^ robotic stapler (Intuitive Surgical, Inc., Sunnyville, CA, USA) with a blue reload cartridge is introduced (Fig. 1F). Two 4–0 V-Loc™ barbed sutures (Medtronic, Minneapolis, MN, USA) from each side close the remaining defect (Fig. 1G and H) and then the entire suture line is reinforced by two continuous 4 − 0 PDS™ II monofilament sutures (Ethicon, Johnson & Johnson MedTech) to produce the final result (Fig. 1I). A final round of indocyanine green (ICG; Verdye^®^, Diagnostic Green GmbH, Aschheim-Dornach, Germany) is injected to assess the final result (Fig. 1J). All anastomoses were typically made just below the azygos vein and advanced only when required for oncological reasons. All anastomoses were performed by two seasoned upper gastrointestinal surgeons (MvD and EK), who each had for years of experience in robotic foregut at the time of introduction of the anastomotic technique.

### Postoperative management

Postoperative management follows a standardized ERAS^®^ protocol. Patients are extubated in the operating room and transferred to the ICU, then to a specialized surgical unit on POD1. Since may 2023, no routine nasogastric tube is used. The JacksonPratt^®^ drain is disconnected from active suction on POD1 if less than 500 ml production and changed to vacuum ballon and removed one day before discharge if amylase < 50 and output < 500 ml/24 hours as well as absence of lymphatic leak. Pleural drains (which are not standard) are removed on POD 1–2 depending on output and air leakage.

Analgesia is provided via a paravertebral catheter plus IV morphine PCA. During the participation in the PEPMEN study (December 2019 to February 2023), included patients were randomized to either paravertebral catheter or thoracic epidural catheter [12].

Patients begin with 500 ml thin liquid oral intake on POD1, advance to 500/1000 ml thick fluids + unrestricted thin liquids by POD2/3, and to unlimited thick fluids and pureed warm meals by POD4. Soft/solid foods from POD 15.

Planned discharge is on POD5 (since May 2023) and before that it was on POD7. Discharge typically occurs to the patient’s home and only in exceptionally frail cases to interim rehabilitation facilities.

## Results

A total of 350 consecutive patients underwent RAMIE-IL using the linear-stapled side-to-side anastomosis. Baseline characteristics are shown in Table 1. The mean age was 66 years, 70% were male, and 44% had an ASA score ≥ 3. Most tumors were adenocarcinomas (86%) and located in the distal esophagus or gastroesophageal junction. Neoadjuvant therapy was administered to 96% of patients.

**Fig. 2:**
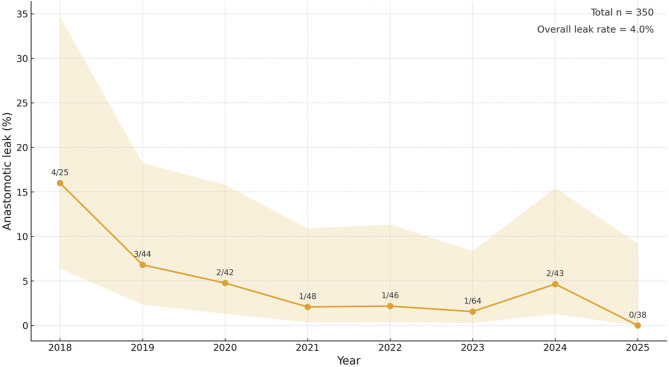
Anastomotic leak rates per calendar year with Wilson 95% confidence intervals

**Fig. 3:**
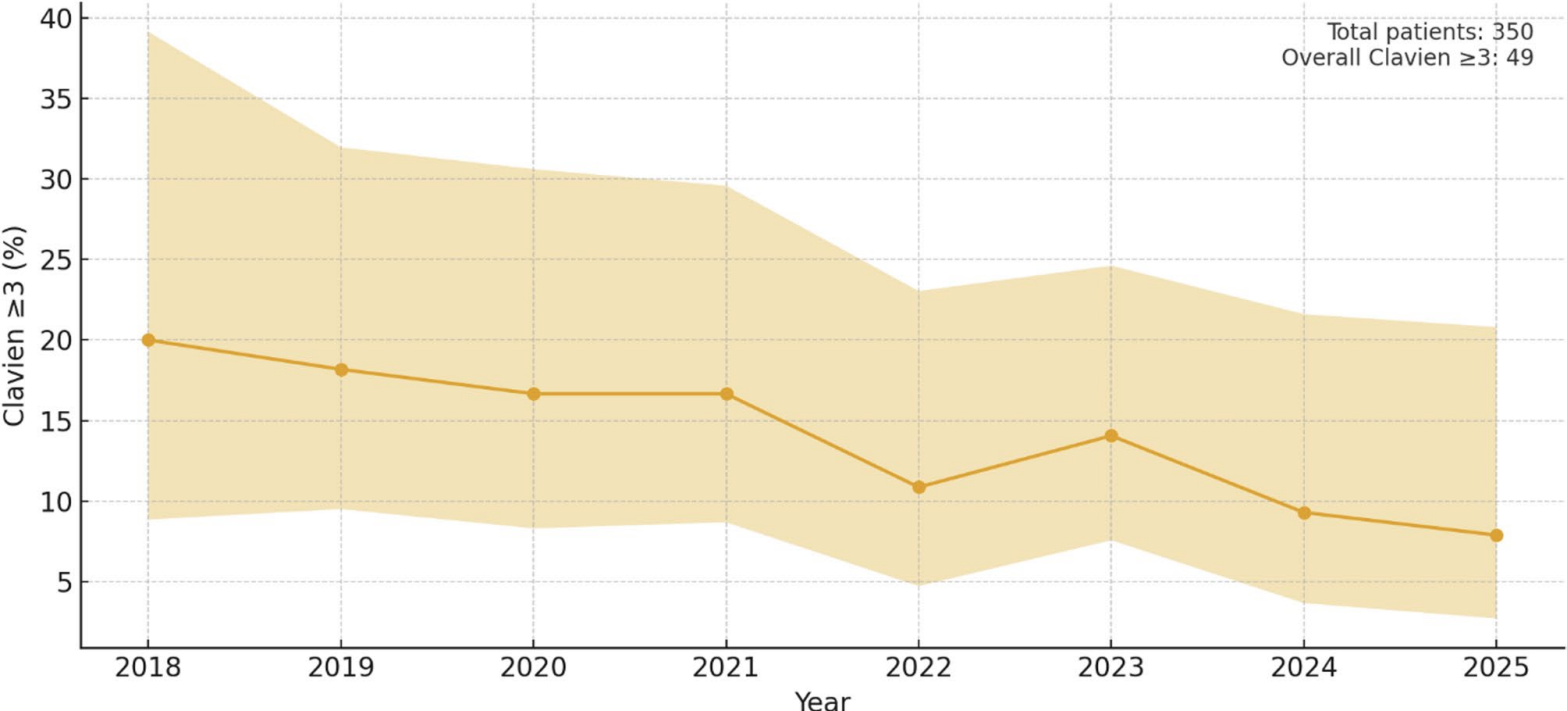
Overall Clavien *≥* 3 rates per calendar year with Wilson 95% confidence intervals

**Fig. 4:**
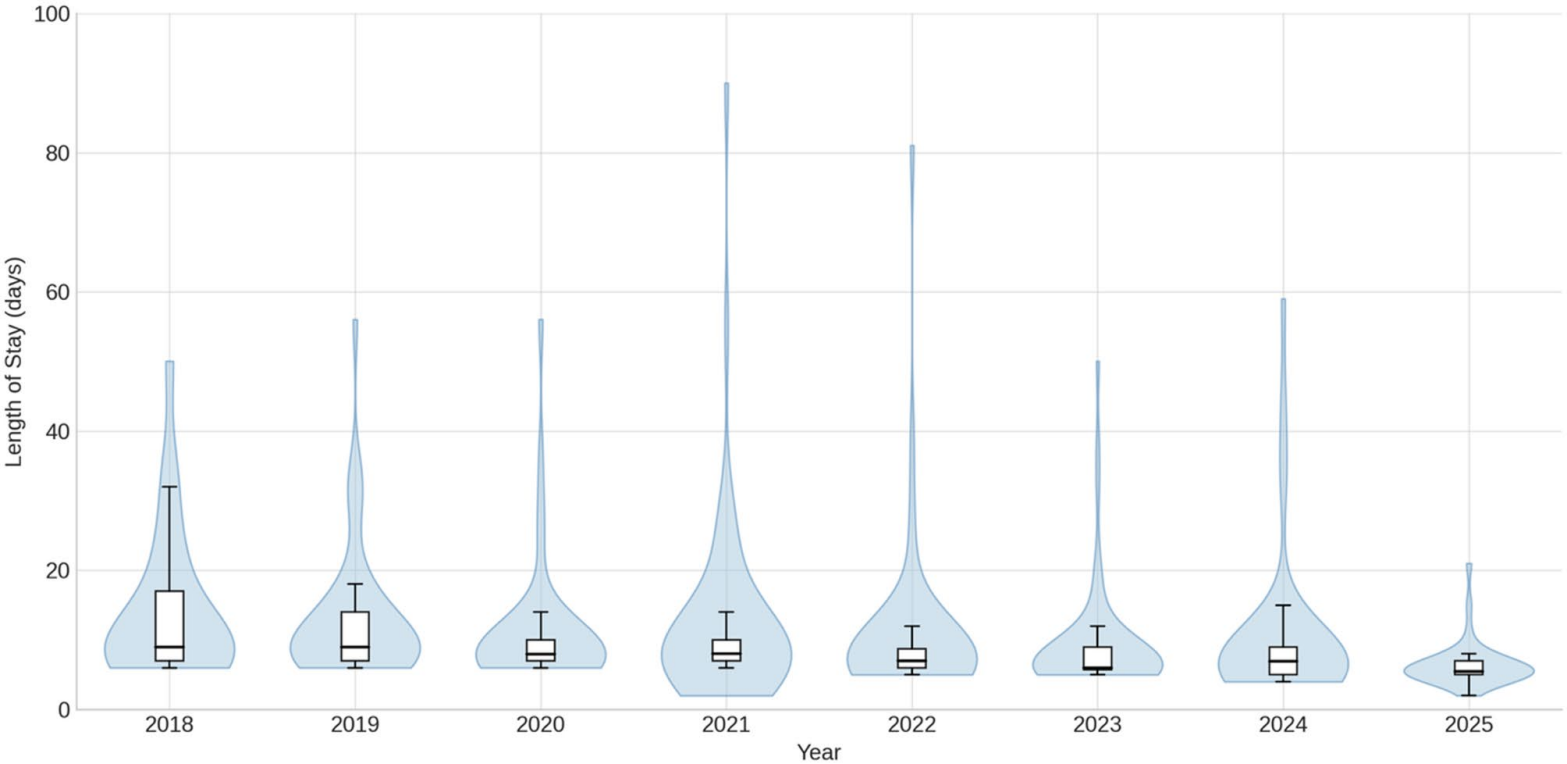
Violin plots with boxplots of the length of stay (LOS) over the study period, sorted per calendar year

Postoperative outcomes are demonstrated in Table 2; Figs. 2, 3 and 4. Overall median length of stay (LOS) was 7 days [IQR 4] and decreased steadily from a median of 9, 9, 8 and 8 days during the first four years to 7, 6, 7, and 5 days during the last four years. ICU stay remained consistently short, with a median of 1 day throughout the study period. The 30-day readmission rate was 12.5% overall. The overall 30-day mortality rate was 1.7%, with no clear temporal trend. Overall major complications (Clavien–Dindo grade *≥* 3) occurred in 14% and decreased steadily over time. The overall anastomotic leak rate was 4%, comprising ECCG type I (*n* = 1), type II (*n* = 9), and type III (*n* = 4) leaks, also steadily decreasing over time. Overall textbook outcome rate was 66%.

**Table 1:** Baseline and clinical tumor data

Age, years mean (±SD)	66 (±9.22)
Gender, n (%)	
Male	244 (69.7)
Female	105 (30)
N/A	1 (0.3)
BMI, kg/m^2^ mean (±SD)	26.41 (±5.9)
Weight loss^*^, kg median [IQR]	4.5 [8.52]
ASA	
I	5 (1.4)
II	191 (54.6)
III	142 (40.6)
IV	12 (3.4)
Histology, n (%)	
Adenocarcinoma	301 (86)
Squamous cell carcinoma	35 (10)
Other	10 (3.5)
Not applicable	3 (0.9)
Unknown	1 (0.3)
Tumor location, n (%)	
Mid	26 (7.4)
Distal	268 (76.6)
GE junction	28 (8)
N/A	28 (8)
Neoadjuvant treatment, n (%)	
Yes	335 (95.7)
No	13 (3.7)
N/A	2 (0.6)
Type of neoadjuvant treatment (*n* = 335), n (%)	
Radiotherapy	28 (8.4)
Chemoradiotherapy (CROSS)	307 (91.6)
cT, n (%)	
T1	8 (2.3)
T2	90 (25.7)
T3	234 (66.9)
T4	7 (2)
N/A	11 (3.1)
cN, n (%)	
N+	229 (65.4)
N-	110 (31.4)
N/A	11 (3.1)

^*^= defined as patient-reported weight loss at the time of the first consultation

**Table 2:** Outcome data

	2018 (*n* = 25)	2019 (*n* = 44)	2020 (*n* = 42)	2021 (*n* = 48)	2022 (*n* = 46)	2023 (*n* = 64)	2024 (*n* = 43)	2025 (*n* = 38)	Overall (*n* = 350)
LOS, days median [IQR]	9 [10]	9 [7]	8 [3]	8 [3]	7 [2.75]	6 [3.25]	7 [4]	5 [2]	7 [4]
ICU stay, days median [IQR]	1 [2]	1 [2]	1 [0]	1 [0]	1 [0]	1 [0]	1 [0]	1 [0]	1 [0]
Readmission within 30 days, n (%)	3 (12)	5 (11.6)	9 (22)	3 (6.3)	4 (8.7)	10 (15.9)	5 (10.5)	4 (11.4)	43 (12.3)
30-day mortality, n (%)	0	1 (2.3)	1 (2.4)	1 (2.1)	1 (2.2)	1 (1.6)	1 (2.3)	0	6 (1.7)
Clavien **≥** 3, n (%)	5 (20)	8 (18.2)	7 (16.7)	8 (16.7)	5 (10.9)	9 (14.1)	4 (9.3)	3 (8.1)	49 (14)
Anastomotic leak, n (%) ECCG 1 ECCG 2 ECCG 3 All	0 3 1 4 (16)	0 3 0 3 (6.8)	1 1 0 2 (4.8)	0 1 0 1 (2.1)	0 1 1 1 (2.2)	0 0 1 1 (1.6)	0 1 1 2 (4.7)	0 0 0 0	1 9 4 14 (4)
Textbook outcome, n (%)	14 (56)	30 (68)	25 (60)	32 (67)	31 (67)	39 (61)	32 (74)	27 (71)	230 (66)

## Discussion

In this large single-center cohort of 350 consecutive RAMIE-IL procedures, implementation of a standardized linear-stapled side-to-side intrathoracic anastomosis was associated with favorable postoperative outcomes and a low overall leak rate of 4%. These results compare favorably with previously reported rates for both minimally invasive and robot assisted Ivor Lewis esophagectomy, where anastomotic leak rates typically range between 5% and 15%^3–7^. The observed differences relative to, for example, the anastomotic comparison performed by the UGIRA (Upper GI International Robotic Association) network are likely largely attributable to differences in study design, case mix and observational nature of the analyses [3]. Nevertheless, these findings are consistent with emerging evidence suggesting lower rates of anastomotic complications in linear-stapled side-to-side anastomoses compared to hand-sewn and circular-stapled techniques [3].

Although causality cannot be proven in an observational design, some temporal trends with improved short-term outcomes are notable.

Both major morbidity and LOS steadily decreased throughout the study period reaching their lowest levels in the last years. These improvements likely reflect several parallel developments, including increasing surgeon experience, progressive refinement of the standardized anastomotic steps and the surgery as a whole, as well as ongoing enhancements, adherence and maturation in perioperative care to ERAS^®^ protocols. Nevertheless, compared to our historic outcomes, complication rates, and particularly anastomotic leaks, decreased substantially after the introduction of the linear-stapled technique. Especially given that the surgical team remained unchanged, no noteworthy changes were made in patient selection criteria, prehabilitation, or neoadjuvant therapy protocols (CROSS regimen, and that all anastomoses were consistently made above the radiation field throughout the study period, the observed improvements appear to be associated with the described anastomotic technique. However, some potential confounding factors merit consideration, particularly participation in the PEPMEN trial and changes in ERAS^®^ protocols, both of which could directly influence postoperative outcomes^13^. Because the PEPMEN trial demonstrated no significant differences in postoperative complications, ICU stay, or total between its intervention groups, we consider it unlikely that participation meaningfully affected the outcomes of the present analysis. In contrast, the update of our ERAS^®^ program in May 2023—changing planned discharge target from POD 7 to POD 5—likely contributed significantly to the observed reduction in LOS. Notably however, LOS was already trending downward before this change. Moreover, our institution is also a high-volume bariatric center performing more than 500 bariatric procedures annually, a factor previously shown by Janssen et al. to be associated with improved short-term outcomes in upper GI resections^14^. Also, it is very important to mention that robotic foregut surgery had already been performed in our institution for four years when this technique was adopted. This existing expertise in foregut surgery, traditional minimally invasive as well as robotic may have facilitated the rapid and safe adoption of this anastomotic approach.

This study has limitations inherent to single-center observational research. Although the dataset is prospectively collected and includes all consecutive cases, no direct comparisons with other anastomotic methods were possible, and unmeasured confounders within perioperative care may have influenced outcomes. Nevertheless, the large cohort size, adherence to a single standardized technique, objective outcome parameters and completeness of follow-up strengthen the validity of the findings. The high textbook outcome rate also ensures comparability with international quality standards.

Overall, the linear-stapled side-to-side anastomotic technique appears safe, reproducible. These results support its adoption as a standard technique in centers performing robotic esophagectomy, particularly when combined with structured training and optimized perioperative care.

## Supplementary Information

Below is the link to the electronic supplementary material.

Supplementary Material 1

## Data Availability

All data are available upon reasonable request.

## Abbreviations

RAMIE-IL: Robot-assisted minimally invasive Ivor Lewis esophagectomy
DUCA: Dutch upper GI cancer audit
ICU: Intensive care unit
ASA: American society of anesthesiologists
SD: Standard deviation
IQR: Interquartile range
ECCG: Esophagectomy Complications Consensus Group
ICG: Indocyanine green
ERAS®: Enhanced recovery after surgery
POD: Postoperative day
